# Using Power Analysis to Choose the Unit of Randomization, Outcome, and Approach for Subgroup Analysis for a Multilevel Randomized Controlled Clinical Trial to Reduce Disparities in Cardiovascular Health

**DOI:** 10.1007/s11121-024-01673-y

**Published:** 2024-05-20

**Authors:** Kylie K. Harrall, Katherine A. Sauder, Deborah H. Glueck, Elizabeth A. Shenkman, Keith E. Muller

**Affiliations:** 1 Department of Health Outcomes and Biomedical Informatics, University of Florida College of Medicine, 2004 Mowry Road, Gainesville, 32606 FL USA; 2 Department of Implementation Science, Wake Forest University School of Medicine, 475 Vine Street, Winston-Salem, 27101 NC USA; 3 Department of Pediatrics, University of Colorado School of Medicine, 13123 E. 16th Ave., Aurora, 80045 CO USA

**Keywords:** Power, Multilevel randomized controlled trial, Composite outcome, Subgroup analysis, Heterogeneity of treatment effect

## Abstract

We give examples of three features in the design of randomized controlled clinical trials which can increase power and thus decrease sample size and costs. We consider an example multilevel trial with several levels of clustering. For a fixed number of independent sampling units, we show that power can vary widely with the choice of the level of randomization. We demonstrate that power and interpretability can improve by testing a multivariate outcome rather than an unweighted composite outcome. Finally, we show that using a pooled analytic approach, which analyzes data for all subgroups in a single model, improves power for testing the intervention effect compared to a stratified analysis, which analyzes data for each subgroup in a separate model. The power results are computed for a proposed prevention research study. The trial plans to randomize adults to either telehealth (intervention) or in-person treatment (control) to reduce cardiovascular risk factors. The trial outcomes will be measures of the Essential Eight, a set of scores for cardiovascular health developed by the American Heart Association which can be combined into a single composite score. The proposed trial is a multilevel study, with outcomes measured on participants, participants treated by the same provider, providers nested within clinics, and clinics nested within hospitals. Investigators suspect that the intervention effect will be greater in rural participants, who live farther from clinics than urban participants. The results use published, exact analytic methods for power calculations with continuous outcomes. We provide example code for power analyses using validated software.

**Supplementary Information:**

The online version contains supplementary material available at 10.1007/s11121-024-01673-y.

## Introduction

This manuscript provides a discussion about three design choices that can increase power for randomized controlled trials with multiple levels of clustering. A Glossary provides definitions for many terms used in the text.

First, we demonstrate that, for a fixed number of independent sampling units, power depends on the choice of level of randomization. Second, we show that testing a multivariate hypothesis, rather than a hypothesis about an unweighted composite outcome, can increase power and interpretability. Finally, we show that using a pooled analytic approach, which analyzes data for all subgroups in a single model, can improve power compared to a stratified analysis, which analyzes data for each subgroup in a separate model (Buckley et al., 2017). The goal of the manuscript is to allow designers facing similar design questions to conduct careful power calculations during the design of their trials.

The design questions are answered for a specific example study. The study is a proposed multilevel randomized controlled prevention research trial. The trial will be an extension of the Agency for Healthcare Research and Quality (AHRQ)-funded observational study R01HS028283 (E. A. Shenkman PI). In the proposed trial, the investigators plan to randomize adults to either telehealth or in person treatment. The goal is to reduce cardiovascular risk factors. The trial outcomes will be measures of the Essential Eight (Lloyd-Jones et al., 2022), an approach to quantify cardiovascular health developed by the American Heart Association. The Essential Eight includes definitions for scores for diet, physical activity, nicotine exposure, sleep health, body mass index, blood lipids, blood glucose, and blood pressure. The scores can be combined into a single unweighted composite score by averaging the domain-specific scores, as suggested on page e28 in Lloyd-Jones et al. (2022). The proposed trial will have multiple levels of clustering, with outcomes measured on participants, participants treated by the same provider, providers nested within clinics, and clinics nested within hospitals. The number of independent sampling units, the hospitals, will be fixed by design. Investigators suspect risk reduction will be higher in rural participants, who live farther from clinics than urban participants.

The proposed trial will have two goals. The first goal will be to assess the effect of intervention on cardiovascular health. The second goal will be to determine whether there were differences in intervention effects between subgroups. Subgroups will be defined by the location of hospitals in rural or urban areas.

During the design process for the proposed trial, three questions about design were raised.

- First, researchers wondered whether to randomize participants, providers, clinics, or hospitals. The researchers were curious about the implication of the choice of level of randomization on power.
- Second, researchers were curious whether testing intervention effects on the unweighted composite outcome would yield more power than assessing whether there was a multivariate response. An unweighted composite score is the sum of a set of variables. A weighted composite multiplies each element of the set of variables by a number, called a weight, and then sums the results. A multivariate response corresponds to an intervention-related change in at least one of the Essential Eight component scores.
- Third, researchers were not certain as to the best way to analyze potential differences in intervention effects between hospitals in rural or urban regions. Some researchers wished to split the data into two sets, one with rural hospitals only and the other with urban hospitals only. These researchers planned to assess intervention effects in each subgroup using separate models. Other researchers argued that estimating intervention effects in both subgroups in the same model was more efficient. Researchers questioned which analytic approach provided the most power. Answering the question required comparing power calculations for two different approaches for analyzing data with subgroups.

To answer the design questions, the investigators for the proposed trial iteratively considered the effect on power of several design alternatives. The researchers worked with statisticians to evaluate the effect of analytic strategies and choices of outcome variables. As in all clinical trials, power was only one of multiple considerations driving design decisions. Researchers also considered costs, ethics, feasibility of recruitment, noncompliance, and the chance of contamination, an event which occurs when those randomized to one intervention receive a different intervention. Multilevel trials are particularly subject to contamination, because participants often share the same classroom, clinic, or other grouping and thus may hear about or participate in a different intervention condition. Costs, ethics, feasibility of recruitment, noncompliance, and the chance of contamination are discussed in depth in other publications, e.g., Cuzick et al. (1997). The goal of this manuscript was to provide information about how to conduct power calculations for three specific design questions which arise in multilevel clinical trials.

Several simplifying assumptions were made. The example uses the Essential Eight as a measure of cardiovascular health. The scoring suggested by Lloyd-Jones et al. (2022) produces continuous outcomes, with a Gaussian (normal) distribution rather than a binary (yes/no), count, negative binomial, or right truncated distribution. For simplicity, the cluster size at each level is assumed to be the same. With balanced cluster sizes at each level, cluster means of continuous data have an asymptotically normal distribution. In turn, the distribution of the test statistic is known exactly, and the test achieves the claimed Type I error rate. No missing outcome data is allowed for any participant. The intraclass correlation coefficient, a measure of correlation between elements in a cluster, is assumed to be the same for each cluster element within a single level and across intervention assignments and subgroups. No covariates are used. The researchers planned to use a general linear mixed model (Laird & Ware, 1982) rather than generalized estimating equations.

The nomenclature used in this manuscript for describing the structure of factors in randomized controlled clinical trials follows a commonly used convention, which describes both nesting and clustering, as used in Moerbeek (2005), Heo and Leon (2008), and Moerbeek and Teerenstra (2015). For the example described above, outcomes are measured on participants, participants are treated by the same provider, providers work together in clinics, and clinics are located within hospitals. Outcomes are nested within participants. Participants are nested within provider. Providers are nested within clinics. Clinics are nested within hospitals. The design thus has four levels of nesting. Because participants are nested within a provider, it is a reasonable assumption that they will have correlated outcomes. A similar assumption holds for providers in a clinic, and clinics in a hospital. Thus, the design has three levels of clustering.

**Fig. 1:**
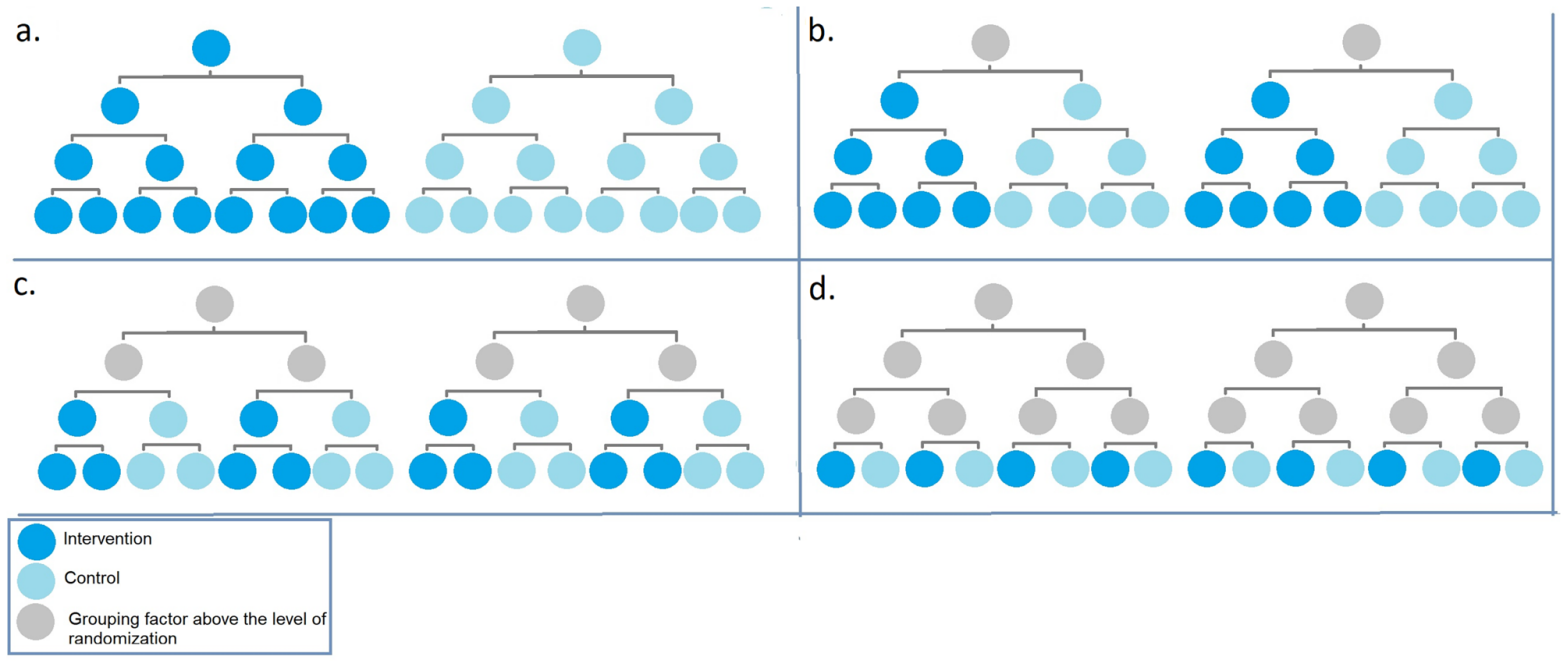
Four levels of randomization in a multilevel trial with four levels of nesting and three levels of clustering (**a** hospital level randomization; **b** clinic-level randomization; **c** provider-level randomization; and **d** study participant-level randomization). For ease of display in the figure, only two independent sampling units, the hospitals, are shown, with two clinics in each hospital, two providers in each clinic, and two study participants per provider. The power calculations have more study participants, providers, clinics, and hospitals. Intervention is shown in azure, with control in Alice blue. Grouping factors above the level of randomization are shown in gray

In a multilevel trial, randomization can occur at the level of the independent sampling unit or within independent sampling units at any level. This is shown schematically for the example study in Fig. 1. For clarity, although the example study will include multiple hospitals, clinics, providers, and participants, the figure shows only two hospitals, two clinics per hospital, two providers per clinic, and two participants per provider. Hospitals appear at the top level. Within each hospital, at the next level, are two clinics. Within each clinic, at the next level, are two providers. Finally, within each provider, at the bottom level, are two participants. Hospitals are considered to be independent sampling units.

There are many possible randomization schemes for designs with clusters. The unit of randomization is the name given to the element of a study design which is randomized. The unit of randomization and the independent sampling unit may be the same, or they may be different, depending on the study design. The difference between unit of randomization and independent sampling unit can be seen in two types of studies with a single level of clustering: the randomized complete block design and the group-randomized design. In a randomized complete block design, randomization occurs within independent clusters. In a group-randomized trial, there is a single level of grouping or clustering, and each independent group or cluster is randomly assigned to an intervention. For group-randomized trials, the independent sampling unit is the same as the unit of randomization.

For designs with multiple levels of clustering, there are many possible ways to conduct randomization. Figure 1 shows four possible levels of randomization for the example multilevel trial. In panel a, the randomization is at the level of the hospital. The entire hospital is randomized to intervention or control. The randomization is *between* independent sampling units. In panel b, the randomization is at the level of the clinic. It is a randomization *within* independent sampling units. In panel c, the randomization is at the level of the provider. Again, the randomization is *within* the independent sampling units. Finally, in panel d, the randomization is at the level of the participant, *within* the independent sampling units. Gray circles above the level of randomization show how grouping or clustering still creates correlation, no matter where the randomization occurs.

This manuscript provides example power analyses for a single example trial for three design choices: level of randomization, form of the outcome, and analytic approach for assessing subgroup differences. A literature review provides background on power and sample size software and methodology for randomized clinical control trials with multiple levels of clustering. The results section provides power curves for each of the three design choices. The discussion section summarizes limitations and strengths of the approach and outlines implications for future research. A detailed mathematical presentation of the predictors, outcomes, covariance structure, models, and hypotheses tests appears in Online Resource 1.

## Methods

### Software Review Methods

The introduction section lists assumptions made for the example. The assumptions led to exclusion and inclusion criteria for a review of software packages. The goal of the literature review was to find software which could be used for the power analyses. Software packages had to compute power for the general linear mixed model, with Gaussian outcomes. Packages designed for use for binary, count, or right truncated outcomes were excluded. Packages designed for computing power for generalized estimating equations were also excluded.

The review of software packages was not an exhaustive or structured literature review. Both Google Scholar and PubMed were searched, using the keywords “multilevel,” “cluster,” and “power” with a Boolean “or”.

The resulting website hits were examined in order of relevance, until the same package appeared in both searches. A Ph.D. statistician (KEM) downloaded the software and read the accompanying documentation. He also reviewed peer-reviewed publications on the software packages, if available, published analytic methods or both, to obtain more detail. Software packages were then judged as being able to compute power for the three design choices.

### Power Methods

The power calculations presented in this study use published analytic power results (Chi et al., 2019) for the Wald test in the mixed model (Laird & Ware, 1982). The calculations were implemented in POWERLIB, a free, validated, open-source SAS $/$ IML package (SAS Institute Inc, 2020) described in Johnson et al. (2009). POWERLIB was chosen because it has the ability to address all three design choices considered in this study. POWERLIB was initially designed to provide power calculations only for the general linear multivariate model. Chi et al. (2019) showed that under certain conditions, the mixed model Wald test with Kenward and Roger (2009) degrees of freedom coincides with the Hotelling-Lawley trace statistic for the general linear multivariate model using the McKeon (1974) reference distribution. Designs with clusters fit the restrictions if (1) all the clusters at each level are the same size, (2) there are no repeated measurements of predictors over time, and (3) there are no missing data in the predictors or outcomes. Under the restrictions, exact or very accurate power approximations for the mixed model can be calculated using results presented by Muller et al. (1992). For the examples and assumptions considered in this manuscript, the results are exact.

The power calculations require seven inputs: the Type I error rate, the covariance of the errors, the between-independent sampling unit contrast, the within-independent sampling unit contrast, the population values of the parameters for a specified alternative hypothesis, the corresponding values for the null hypothesis, and the sample size in each group. Inputs for the power calculations shown in this paper appear in Online Resource 1. Power curves were produced from the points generated from POWERLIB using SAS SGPLOT. Approximately 100 power values were calculated for each power curve in order to accurately reflect the smoothness of the underlying function.

Glossary **Contamination**

The occurrence, in a randomized controlled clinical trial, of an event in which a cluster, independent sampling unit, or both, are exposed to an intervention other than that assigned by the randomization scheme.

**Cluster**

A group, with membership determined by common exposures, experiences, background, or interactions among group members.

**Composite**

A variable formed by summing or averaging the possibly weighted values of other variables.

**Exchangeability**

An assumption about the joint distribution of a set of random variables, each of which has a number, which indicates that the joint distribution does not change if the variables are renumbered. In cluster sampling, the assumption of exchangeability leads to elements of a cluster having equal variance and equal correlation.

**Intraclass Correlation Coefficient or ICC**

The common correlation among any two items due to a single level of clustering.

**Independent Sampling Unit**

Generically, a unit in a study design which is statistically independent of all other units of the same sort. In a multilevel study, a group or cluster which is statistically independent from all other groups or clusters at the same level. Often, the level in a multilevel trial in which all the other levels are nested, but that itself is not nested in another level.

**Multilevel**

A study involving data structures with more than one dimension of grouping or clustering, often with clusters nested within clusters. Here, multilevel indicates multiple levels in a hierarchical design, rather than describing a multilevel intervention, which is applied at many levels of a study.

**Nesting**

A feature of study designs which occurs when levels of one factor appear only within levels of another factor.

**Power**

The probability of rejecting the null hypothesis, evaluated for a particular alternative hypothesis.

**Treatment Effect Heterogeneity**

Differences in the effect of intervention between subgroups.

**Unit of Randomization**

Generically, the unit in a study design which is randomly assigned to an intervention or control group. In a multilevel trial, it indicates the level at which randomization takes place.

**Unweighted Composite**

A variable formed by summing the unweighted values of other variables.

## Answering the Design Questions

### Assumptions Used for All Design Questions

A power analysis for each design question is described in each of the next three subsections. Each subsection explains the question at hand, describes the hypothesis of interest, the data analysis planned, and the aligned power analysis. An aligned power analysis matches the planned data analysis in terms of model, hypothesis, statistical hypothesis tests, reference distributions, and Type I error rate (Muller et al., 1992). For analysts who want more information, mathematical details appear in Online Resource 1. The Online Resource 1 details matrices defining the predictors, outcomes, models, and between- and within-independent sampling unit hypotheses.

The example power analyses assumed 20 hospitals, 8 clinics per hospital, 6 providers per clinic, and 6 participants per provider. All examples assume that all 8 outcomes are measured on all participants. For the subgroup analysis, it is assumed that there are 10 urban hospitals and 10 rural hospitals. The power analysis also assumed an equal number assigned to intervention and control.

The sampling design of a study dictates the correlation and variance of the outcomes. By convention, single measurements on elements in a cluster are assumed to be exchangeable, which means that their sampling distribution does not change if the elements are renamed or renumbered. In cluster sampling, the assumption of exchangeability leads to elements of a cluster having equal variance and equal correlation. The three-level cluster correlation model (Longford, 1987) assumed the intraclass correlations are 0.05 for provider, 0.01 for clinic, and 0.01 for hospital. Therefore, the hypothesized correlation between any two participants seeing the same provider in the same clinic in the same hospital should be $0.05+0.01+0.01=0.07$.

The variance of the outcomes arises from two sources. One source is the clustering of participants within providers, providers within clinics, and clinics within hospitals. Another source is the covariance between the Essential Eight outcomes. As detailed in Online Resource 1, all models with the multivariate outcomes used a Kronecker product of the three level cluster covariance model and an unstructured covariance model among the eight outcomes for data analysis. In order to simplify the narrative, the power analyses assumed the subscales of the Essential Eight were independent with variances of 1. For models with the unweighted composite outcome, the variance model is a Kronecker product of the three-level cluster covariance model, with the scalar variance of the unweighted composite outcome.

For each design question, researchers planned to fit a general linear mixed model which varied with the level of randomization. The investigators planned to use the Wald test with Kenward and Roger (2009) degrees of freedom at an alpha level of 0.05.

### Design Consideration 1: Level of Randomization

Researchers conducted a power analysis to examine the effect of choice of level of randomization on power. The four randomization schemes considered are shown in Fig. 1. Changing randomization level changes the distributions of the estimates of means, as well as their variances and correlations. Accurate power analysis requires correctly accounting for the differences.

Researchers sought to examine the effect of intervention on a single, unweighted composite outcome, formed by averaging the Essential Eight outcomes. The scientific question was whether intervention would alter the average response for the unweighted outcome more for those randomized to intervention, compared to control. The null hypothesis was that there would be no intervention effect on the unweighted composite outcome. The alternative hypothesis was that the composite outcome would change, as a result of the intervention effect.

Any power calculation computes power for a specified pattern of results. In most cases, there are a variety of patterns which are plausible. In this case, the researchers computed power supposing that intervention caused a change in only one of the eight outcomes, thinking that the intervention would change diet, but no other of the Essential Eight.

### Design Consideration 2: Unweighted Composite Versus Multivariate

Researchers conducted a power analysis to examine the effect of the choice of outcome type on power. Randomization was assumed to occur at the level of the independent sampling unit, the hospital (see Fig. 1a).

The scientific question for the multivariate outcome is whether intervention has an effect on any of the Essential Eight outcomes. The grand null hypothesis is that there is no difference in any of the eight subscores between those randomized to intervention or control. It is a grand null, rather than a single null, because it involves the combination of eight hypotheses. The alternative hypothesis is that there is at least one mean subscore which differs between those randomized to intervention or control.

The scientific question for the composite outcome is whether intervention has an effect on the average Essential Eight. The null hypothesis is that there would be no effect of intervention on the composite outcome. The alternative hypothesis was that there would be an intervention effect on the composite outcome.

The researchers considered two mixed models: one with a multivariate outcome and one with an unweighted composite outcome. Both models included fixed effect predictors for an intercept and an indicator variable for treatment. The variance models are described above.

The specific alternative considered for both power analyses was that there was exactly one subscore which differed between the intervention and control groups. As we shall note in the Discussion, power depends on the alternative chosen. The power calculations would certainly change if more subscores differed between the intervention and control groups.

### Design Consideration 3: Stratified Versus Pooled Subgroup Analysis

We considered two approaches for analyzing data with subgroups. A *stratified* approach, described by Buckley et al. (2017), splits the example trial data into two separate sets, one with rural hospitals and the other with urban hospitals. Modeling and hypothesis testing are reported separately for each set. If the subgroups are of equal size, the separate sets each have half the number of independent sampling units as the original data set. The stratified analysis has two hypothesis tests. In each subset, the investigators planned to test separately the null hypothesis that, for the subgroup considered, there is no difference in the average of the single outcome between those randomized to intervention or control. The alternative hypothesis assumed is that, for the subgroup considered, there is some difference in the average of the single outcome for those randomized to intervention or control.

A *pooled* analysis conducts modeling and hypothesis testing in the full data set. The approach was described in Muller and Fetterman (2003). They recommended the pooled approach because it generally has more power. In the pooled analysis, investigators planned to test the null hypothesis that there is no difference in the average of the single outcome between those randomized to intervention or control, averaged over subgroup. The alternative hypothesis is that there was a difference in the average of the single outcome between those randomized to intervention or control, averaging over subgroup. The hypothesis test is conducted under the implicit assumption that there is no difference in treatment effects between the subgroups.

For both the stratified and pooled analyses, the researcher fit a general linear mixed model, with the unweighted composite as the outcome. For the pooled analysis, the fixed effect predictors included an intercept, an indicator variable for intervention, an indicator variable for subgroup, and the subgroup-by-intervention interaction. The approach was adopted because of the belief that including the effect modifier (the interaction term) generally leads to an efficiency gain, at least in single level trials as shown by Tong et al. (2022) and Tong et al. (2023). For the stratified analysis, the fixed effect predictors included an intercept and an indicator variable for subgroup. The covariance model was as described above. Estimates of mean effect were computed for the urban hospitals randomized to intervention, the urban hospitals randomized to control, the rural hospitals randomized to intervention, and the rural hospitals randomized to control. Variances were estimated in the entire sample.

Power for the pooled approach was computed similarly as for the stratified approach. One additional assumption was made. Power is always computed for a specified alternative hypothesis. The approach adopted here was to assume that there was no interaction. The power computation thus assumed that the interaction parameter was included in the model, but that interaction term was zero.

An alternative power calculation would have been to consider a model with no fixed effect interaction term and to compute power for the intervention effect in that model. Because each fixed effect in the model reduces the error degrees of freedom by one, the approach we chose is slightly more conservative.

## Results

### Software Review

A literature review of current methods and software for power analysis showed that only one power calculation method, and associated software package, provides power computations for all three design choices considered, under the restrictive assumptions about design, model, hypothesis test, and reference distribution. A summary of the review appears in Table 1. Software packages were included in the table if they could compute power for trials with four or more levels of nesting and three or more levels of clustering. Most existing software packages lacked applicability to at least one of the three cases discussed in this paper. Most packages provide results for single outcomes, but do not account for both multilevel design and multiple outcomes. In many software packages, comparison of power at different levels of randomization was not straightforward. Some packages could not provide power for subgroup factors.

**Table 1:** Review of methods and software for power and sample size for multilevel randomized controlled clinical trials or observational studies with at least four levels of nesting and three levels of clustering

Software package	Reference	Levels of clustering	Choice of level of randomization within independent sampling unit	Subgroup factor	Composite versus multivariate outcomes
CRT-POWER	Borenstein et al. (2023)	3	$\checkmark$		
GLIMMPSE	Kreidler et al. (2013)	$>3$		$\checkmark$	$\checkmark$
Optimal Design	Raudenbush et al. (2011)	3	$\checkmark$		
POWERLIB	Johnson et al. (2009)	$>3$	$\checkmark$	$\checkmark$	$\checkmark$
PowerUp	Dong and Maynard (2013)	2			
SPA-ML	Moerbeek and Teerenstra (2015)	2			

To arrive at the set of software packages included in Table 1, we reviewed many other power and sample size software packages. Specific examples of packages limited to two or fewer levels of nesting included the Research Methods Resources (National Institutes of Health, 2022) and a variety of R packages, such as Kleinman et al. (2021). The Mplus package (Muthén & Muthén, 2023) allows specifying a data analysis and conducting a simulation for a specific design in order to assess power. Finch and Bolin (2016) described how to allow up to three more levels of nesting and up to two levels of clustering in Mplus. The commercial software packages NCSS$/$PASS (NCSS, 2008), SAS (SAS Institute Inc, 2011), and STATA (StataCorp, 1985) have power methods that explicitly account for only one level of clustering (two levels of nesting).

Several manuscripts were found, some with accompanying software, which provided power for analytic approaches or outcomes other than that considered in this manuscript. A general review appears in Turner et al. (2017). Subsequently, Li et al. (2019) discussed power and sample size methods for analysis of cluster-randomized cross-over trials with generalized estimating equations. Wang et al. (2022) provided power for generalized estimating equations for four level cluster-randomized trials. Candel and Van Breukelen (2010) provided sample size for varying cluster sizes in cluster randomized trials with binary outcomes. The relative efficiency of cluster randomized designs or multicenter trials with equal or unequal cluster sizes was discussed by van Breukelen et al. (2007). Tong et al. (2022) also discussed unequal cluster sizes in analyses of treatment effect heterogeneity.

### Power Results

**Fig. 2:**
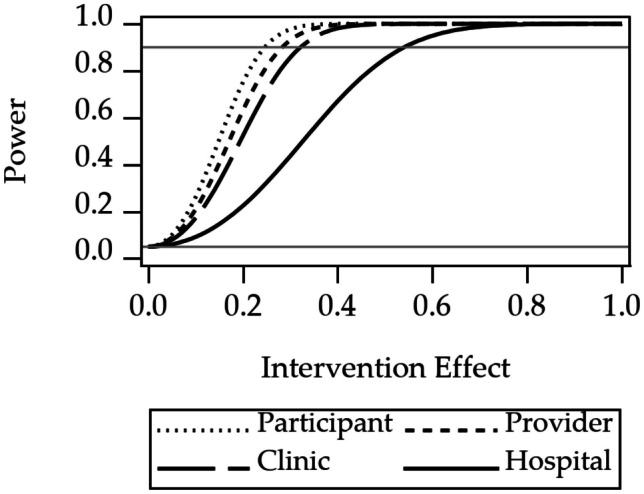
Power for randomization at level of study participant, provider, clinic, or hospital. The gray reference line at 0.05 is the Type I error rate, a lower limit for power. The gray reference line at 0.9 indicates a common target power for clinical trial design

**Fig. 3:**
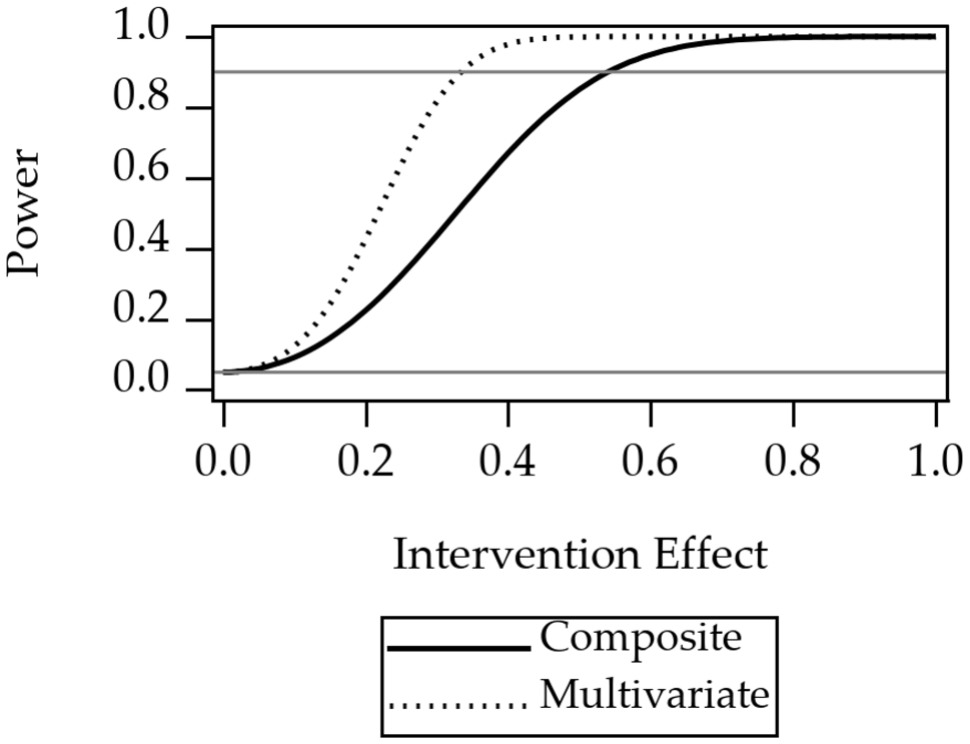
Power for unweighted composite outcome versus multivariate outcome. The 1.0 on the horizontal axis corresponds to a difference between the intervention and the control groups of one standard deviation of one of the Essential Eight variables. The gray reference line at 0.05 is the Type I error rate, a lower limit for power. The gray reference line at 0.9 indicates a common target power for clinical trial design

**Fig. 4:**
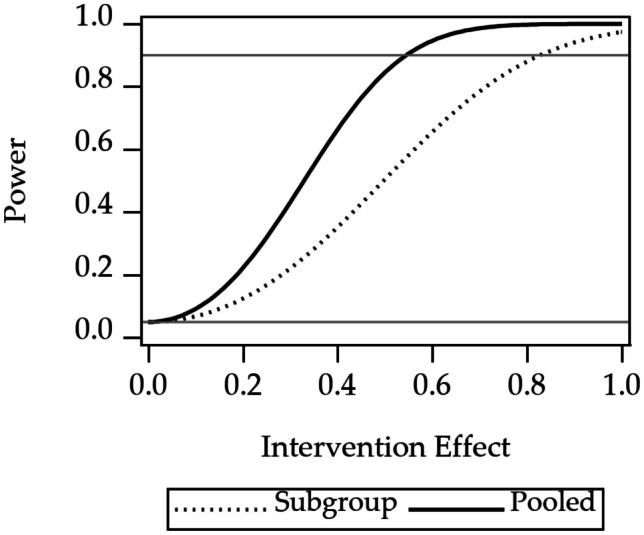
Power for stratified analysis of subgroups, versus pooled analysis, for a single outcome variable of the Essential Eight. There are two subgroups, so the sample size for the stratified analysis is half that of the pooled analysis. The 1.0 on the horizontal axis corresponds to a difference between the intervention and the control groups of one standard deviation of one of the Essential Eight variables. The gray reference line at 0.05 is the Type I error rate, a lower limit for power. The gray reference line at 0.9 indicates a common target power for clinical trial design

Power curves for the three design considerations are shown in Figs. 2, 3, and 4. Power curves for randomization at one of four levels of nesting of the example multilevel study are shown in Fig. 2. The curves show that power is highest for randomization at the level of participant. Power decreases when moving to an outer level of clustering. Power curves for an unweighted composite versus multivariate outcomes are shown in Fig. 3. The curve shows that power for the multivariate approach is higher than the power for the composite outcome. Power curves for stratified versus pooled modeling to assess treatment are shown in Fig. 4. Because the sample size for the stratified models is half that of the sample size for the pooled approach, the power is higher for the pooled approach.

Code to create the power curves appears in Online Resource 2, with an index to the files in Index.txt. Online Resource 2 contains programs (.sas), code modules (.iml), associated data (.sas7bdat), result files (.lst), and plots (.png). All.sas,.iml, and.lst files are text format.

## Discussion

This manuscript provided a discussion of three questions about the design of a particular multilevel randomized controlled clinical trial. For this example, there were three conclusions. First, for the example considered, the choice of unit of randomization can change the power, with power the highest for the choice of unit of randomization at the lowest level of nesting. For the example, power is highest for randomization of participants, rather than hospitals. Second, for the example considered, using a multivariate hypothesis, rather than a hypothesis about an unweighted composite outcome, can substantially increase power. Third, for the example considered, there can be a substantial gain in power by using a pooled analysis strategy, rather than splitting the sample by subgroup and evaluating the intervention effect in each subgroup-specific sample.

It is our hope that readers will find it useful to have solution strategies to all three design questions in the same place. The manuscript provides a unique discussion about the selection of multivariate versus composite outcomes, illustrating that the best analysis strongly depends on the choice of test and the underlying patterns of data. Our power analyses utilized peer-reviewed validated software (Johnson et al., 2009) and methods, including those of Muller et al. (1992), Muller et al. (2007), and Chi et al. (2019). So that other researchers may replicate our designs, we have provided straightforward and annotated code for each design.

In general, a tenet of power analysis is that higher sample sizes lead to more power. Two of our findings are consistent with this tenet. We showed that randomization of participants, instead of hospitals, increases power. The result occurs because there are more participants than hospitals in the study. We also showed that pooled, rather than stratified, analysis approaches have more power. The result occurs because there are more units of randomization in the pooled analysis.

There are both ethical and economic reasons to conduct careful power calculations and make considered design choices in multilevel randomized controlled clinical trials. Like all randomized controlled clinical trials, multilevel randomized controlled clinical trials can take a huge amount of time and money. Recruiting and retaining participants in a randomized controlled clinical trial require substantial investment by study staff. In addition, any trial with human participants can incur ethical costs. Study participants may experience potential harms from research, in addition to the inconvenience and time costs for participating in a trial. Studies which are too large place more participants than necessary at potential risk. Studies which are too small to have sufficient power place participants at risk for little or no chance of being able to adequately test a hypothesis.

This manuscript considered each design question separately. An alternative approach would have been to consider more complicated designs with combinations of design features, which is typical of actual research. For example, the manuscript could have considered the effects on power of changing both level of randomization, and whether the outcome was composite or multivariate, at the same time. We avoided complex designs in order to make the results easier to understand. It is important to note that the methods used do accommodate any combination of the study design features considered.

The goal of this manuscript was to encourage careful calculation of power during the design of every study. Conclusions from the example considered may not generalize to other designs. The example considered was a design with outcomes measured on participants, participants treated by the same provider, providers nested within clinics, and clinics nested within hospitals. Conclusions for this manuscript only are true for a design which matches this study in terms of sample size, levels of nesting, levels of clustering, number of independent sampling units and clusters, sample size within clusters, intraclass correlations, covariances among outcomes, pattern of means, and statistical test procedure. Specifically, the example design has four levels of nesting, three levels of clustering, certain dimensions, variances, correlations, means, statistical model, statistical test, reference distribution, and null and alternative hypotheses. Changing any feature will change the power values.

In general, multilevel studies require special methods for power and data analysis. Failure to align power and data analysis approaches with the study design can result in misspecification of power, incorrect choices of sample size, and increased rates of decision errors (Muller et al., 1992). There are extensive treatments of analytic approaches for multilevel studies. See, e.g., Verbeke and Molenberghs (2009) and Cheng et al. (2010) for data analysis with the general linear mixed model and Muller and Stewart (2006) for the theory of the general linear univariate, multivariate, and mixed models. Accurate analytic approximate and exact power and sample size calculations are also available for a wide swath of hypotheses using the general linear multivariate model (Muller et al., 1992) or using the general linear mixed model (Muller et al., 2007; Chi et al., 2019).

This paper used the general linear mixed model, the general linear hypothesis, and the Wald test with Kenward and Roger (2009) degrees of freedom. Other authors have suggested instead using generalized estimating equation-based approaches, with data analytic approaches using small sample corrections for small numbers of independent sampling units (Kauermann & Carroll, 2001; Mancl & DeRouen, 2001). Parallel results for binary outcomes are described by Li and Redden (2015).

The number of independent sampling units is often fixed by the funding agency. For example, a multi-center trial funded by the National Institutes of Health may make awards to a certain number of clinical centers. The number is usually determined by the funds available and by the number of clinical centers who submit well-scored grants. When the number of independent sampling units is fixed by design, one reasonable approach to maximize power is by considering the effect of changing the level of randomization.

Statistical and power concerns should only be indulged when changing the level of randomization will not cause ancillary changes to the study results. In some cases, pragmatic considerations must supersede power considerations. One example occurs when contamination is a concern. Contamination occurs when those randomized to one intervention receive a different intervention than planned. Contamination tends to bias the results of a study towards the null when the study is analyzed as randomized. Multilevel studies are particularly subject to contamination, because the close relationship among members of clusters leads to cross-over between randomization arms. For example, participants in a single clinic may discuss their treatment in a waiting room. Those who were randomized to placebo, rather than intervention, may demand breaking the masking and receiving the intervention instead. Thus, both pragmatic implementation questions and power considerations should be considered when deciding on the level of randomization.

This manuscript presents code and mathematical detail for computing power for multivariate or composite outcomes. However, power is just one concern when choosing outcomes. Testing intervention effects on either a multivariate or a composite outcome evaluates different scientific questions. The multivariate hypothesis tests whether there is a difference between randomization groups in any outcome. It is important to note that a rejection of a multivariate test does not guarantee the randomization groups differ for any single outcome, but for some weighted combination of the outcomes. Considering an unweighted composite outcome asks whether the average level of the outcomes differs between the randomization groups. A potential deficit of an unweighted composite outcome is the possibility that an intervention may lead to an increase in some univariate outcomes and a concomitant decrease in other outcomes. If increases match decreases, the combination may lead to a misleading null result. If there is uncertainty about the direction of the effect for one or more univariate outcomes, a composite outcome should be viewed with caution.

Whether a multivariate outcome leads to more power than an unweighted composite depends on the hypotheses tested, the pattern of means, the covariance pattern, and the dimensions of the design. In the scenario considered, the researchers compared two different hypotheses. For the unweighted composite outcome, they planned to test whether those randomized to intervention had different average levels of the unweighted composite outcome. For the multivariate outcome, the researchers planned to test whether there was any difference in any of the univariate outcomes. An unweighted composite can have more power than a multivariate test if the unweighted composite components (1) share a common scale and (2) have equal magnitudes and directions of association with the outcome. In other cases, the unweighted composite can have lower power, as demonstrated by the example. The conclusion can also vary with the choice of covariance pattern among the outcomes.

The study assumed that the investigators wished to examine only intervention effects, not examine interaction. In general, in studies with subgroups and an intervention, investigators can test at least three distinct hypotheses. Researchers may want to test the interaction hypothesis and find out whether the intervention effect differs within subgroups. Researchers may wish to test the intervention main effect and see if randomization to intervention or control affects the outcome, assuming no interaction. Rarely, researchers may want to test the subgroup effect and see if, for example, urban hospitals have different responses than rural hospitals, again assuming no interaction. Because power depends on what hypothesis is of scientific interest, researchers must compute power for the correct hypothesis. In a randomized controlled clinical trial, with an intervention and a subgroup, analysts must decide how to model and test for interaction. Interaction occurs when the intervention has a differential effect within different subgroups.

For a clinical trial with subgroups, a common approach to test differences in intervention effects between subgroups is to use a planned cascade of hypothesis tests (Muller & Fetterman, 2003). First, the subgroup-by-intervention interaction is tested. If the subgroup-by-intervention interaction hypothesis is significant, the hypothesis test result is reported, and further hypothesis testing is used to examine the intervention effects which are distinct within each subgroup. If the subgroup-by-intervention interaction hypothesis is non-significant, the interaction term may be removed from the model. A more conservative approach is to leave the non-significant interaction term in the model and then test the intervention effect.

A different approach for studies with subgroups is to stratify the data and to examine the intervention effect separately within each subgroup (Buckley et al., 2017). We demonstrate that this approach has much less power because it has a much smaller sample size. It also precludes the ability to test for treatment-by-subgroup interaction and to estimate the size of the differences in treatment effect among subgroups. For that reason, we, like Muller and Fetterman (2003), suggest always using the pooled analysis. In particular, because there may be disparities among cardiovascular health among subgroups, it is important to design studies with sufficient power to examine differences in treatment effect.

The study considered in this manuscript had several limitations. First, the study used examples, rather than general mathematical proofs, to show results for one study design, rather than for many study designs. A higher standard of evidence would be to use derivations to obtain closed-form results for multiple study designs. Second, the calculations for the study used POWERLIB (Johnson et al., 2009). Although POWERLIB is open source, it is inaccessible to most scientists, because it requires a paid license for SAS$/$IML (SAS Institute Inc, 2020), and assumes a knowledge of matrices and multivariate linear models sufficient to state the model, alternative, and hypothesis. In addition, the use of POWERLIB requires assumptions about the study design which rarely hold in multilevel studies. Rarely, if ever, are all the clusters at each level the same size. Covariates may be measured repeatedly over time. Missing data is the norm, rather than the exception, in studies in prevention science. However, even under departures from these assumptions, the power results can provide good guidance. Clearly, further work is needed to develop power and sample size methods allowing unequal cluster sizes in complex cluster designs.

The manuscript was focused on a specific example, with three design choices. To simplify the discussion, several restrictive assumptions were made. This meant that the great generality of possible models, analyses, and tests were not considered. The study did not consider computing power for confirmatory analyses of treatment effect heterogeneity (Li et al., 2022). In addition, the example study did not include covariates, which obviated discussion of covariate intraclass correlation (Yang et al., 2020).

In addition, the study considered only one possible alternative hypothesis for the comparison of power for unweighted composite versus multivariate outcomes. Power depends strongly on the alternative of interest. For this reason, we urge analysts to compute power for all plausible alternatives for their study design. In addition, the study only considered one analytic alternative to examining intervention effects on an unweighted composite outcome, i.e., using a multivariate test. It is possible that a gated analytic approach such as that suggested by Shaffer (1986) or separate Bonferroni-corrected tests (Bonferroni, 1936) for each outcome would perform better. Using the Bonferroni-corrected tests corresponds to considering a joint set of univariate hypotheses. Each hypothesis states that there is no intervention effect for a particular outcome, out of the set of eight outcomes. This approach is typically used if the univariate outcomes are co-primary.

The power calculation used standardized inputs and made the assumption that subscales for the Essential Eight were independent for the power analysis. The assumption is likely inadequate, because outcomes such as smoking, exercise, blood pressure, and lipids are often correlated. POWERLIB does allow computing power for correlated outcomes and accepts the correlation and variance of the error matrix as inputs. However, authors often do not provide sufficient information in publications to obtain this input. Power results differ for independent and non-independent outcomes. Thus, finding better inputs improves power estimates. Harrall et al. (2023) recommended approaches for finding inputs for power and sample size analysis from published articles or existing data. The updated scoring system for the Essential Eight (Lloyd-Jones et al., 2022) has only recently been published, meaning that accurate inputs for power analysis have not been published, to our knowledge, as of this writing. Estimates of variances but not covariances for the Essential Eight subscores did appear in supplemental material for Shetty et al. (2022). The power analyses presented in the present paper assume standard deviations of a single observation for a single participant was 1.0. This approach was critiqued by Lenth (2001) who suggested computing power in actual units of the data to be collected. In general, we strive to follow Lenth’s recommendations, but admit defeat when estimates are not available.

One design question considered in the study was whether a pooled or stratified data analytic approach had more power. The analytic approach used for the pooled analysis included fixed effect predictors for an intercept, an indicator variable for intervention, an indicator variable for the subgroup, and an intervention-by-subgroup interaction term. Power was computed for the main effect of intervention, while setting the interaction parameter to zero. An alternative approach would have been to compute power for the interaction hypothesis. For single-level (rather than multilevel) cluster randomized designs, such power calculations have already been studied by Tong et al. (2022) and Tong et al. (2023).

One goal of this study was to encourage investigators considering multilevel designs to conduct accurate power analysis and consider design alternatives. Power analysis takes time and care to do right and is frequently conducted before funding arrives. However, the results presented here demonstrate that large power differences can occur with what appear to be minor design decisions. The amount of time and money spent on a power analysis will, in retrospect, pale in comparison with the monetary costs and psychic regret engendered by a failed trial.

The study also had several strengths. Use of a published and validated software package (Johnson et al., 2009) based on published power and sample size approximations (Muller et al., 1992) guarantees accuracy of results. The approximations reduced to exact results for all designs evaluated in the present paper. While simulation-based power approximations can be accurate, without industry-standard unit-checking, published validation, and peer-reviewed and publicly available code, simulation results do not meet National Academy of Science standards for reproducibility (National Academies of Sciences, Engineering, & Medicine, 2019). Finally, the study used an approach we have described elsewhere, including in Section 16.4 in Muller et al. (1992) and in Chi et al. (2019). The approach centers on transforming the original models to power equivalent models which simplify the presentation and computations. A power equivalent analysis provides exactly the same power, using a simplified model, hypothesis, or both. As detailed in Online Resource 1, all of the power computations for the study used power equivalent models.

The results of this study show that with increasing study design complexity comes the requirement for more intensive study planning. While more research is needed to generalize the results, to evaluate the utility of different analytic approaches, and to provide analytic results for power at various choices of levels of randomization, this study provides first steps towards the goals. Once the analytic results are published and validated, adding the resulting methods into a free, open-source, validated, point-and-click, wizard-style program such as GLIMMPSE (www.SampleSizeShop.org.), described in Guo et al. (2013) and Kreidler et al. (2013), will provide greater access and dissemination.

### Supplementary Information

Below is the link to the electronic supplementary material.

Supplementary file1 (PDF 257 KB)

Supplementary file2 (ZIP 303 KB)

## Data Availability

No data nor materials were used in this manuscript.

## Code Availability

All code used to generate the results appears in Online Resource 2.
