# Supplementary material for: Using Power Analysis to Choose the Unit of Randomization, Outcome, and Approach for Subgroup Analysis for a Multilevel Randomized Controlled Clinical Trial to Reduce Disparities in Cardiovascular Health
Source: Prev Sci. 2024 May 20;25(Suppl 3):433–45. doi: 10.1007/s11121-024-01673-y (PMC11239604; doi:10.1007/s11121-024-01673-y)
Supplement: Supplementary file 1 — Supplementary file1 (PDF 257 KB) [file 11121_2024_1673_MOESM1_ESM.pdf]

1 Online Resource 1: Power Theory for Example Models and Hypotheses

1.1 Overview and Notation

The following statements of the theory for the general linear mixed and multivariate models uses a unified notation system that avoids notational conflicts. An explicit process is given to transform any multivariate model to a mixed model. For each design question considered, inputs for power analysis are given for a multivariate model and then for a mixed model. A method for defining simpler models that are power equivalent to the original model of interest is outlined.

The (left) direct product of two matrices is written $\boldsymbol{A} \otimes \boldsymbol{B} = \{a_{ij}\boldsymbol{B}\}$. More generally, for a set of square matrices, $\{\boldsymbol{A}_i\}$, the direct sum, written $\boldsymbol{D} = \bigoplus_{i=1}^{N} \boldsymbol{A}_i$, is the block diagonal matrix with the $\{\boldsymbol{A}_i\}$ on the diagonal.

It is common to define the general linear mixed model as a statement about only the observations for independent sampling unit $i \in \{1, \ldots, N\}$ (see Muller and Stewart (2006), Section 6.7). The subscripts m and M distinguish mixed from multivariate model properties. The mixed model for $p_i \times 1$ vector of responses $\boldsymbol{y}_i$ includes fixed-effect predictors $\boldsymbol{X}_{mi}$ ($p_i \times q_m$, fixed and known) and random-effect predictors $\boldsymbol{Z}_i$ ($p_i \times r$, fixed and known):

$$\boldsymbol{y}_i = \boldsymbol{X}_{mi}\boldsymbol{\beta}_m + \boldsymbol{Z}_i\boldsymbol{d}_i + \boldsymbol{\mathfrak{e}}_{mi} \ . \tag{1}$$

Here ($q_m \times 1$) matrix $\boldsymbol{\beta}_m$ contains unknown fixed-effect parameters, and $\boldsymbol{X}_{mi}$ includes both between- and within-hospital design information. The distributions are

$$\begin{bmatrix} \boldsymbol{d}_i \\ \boldsymbol{\mathfrak{e}}_{mi} \end{bmatrix} \sim \mathcal{N}_{r+p_i}\left(\begin{bmatrix} \mathbf{0} \\ \mathbf{0} \end{bmatrix}, \begin{bmatrix} \boldsymbol{\Sigma}_{\boldsymbol{d}} & \mathbf{0} \\ \mathbf{0} & \boldsymbol{\Sigma}_{\boldsymbol{\mathfrak{e}}_{mi}} \end{bmatrix} \right) \ , \tag{2}$$

which implies $\mathbb{E}\left(\boldsymbol{y}_i\right) = \boldsymbol{X}_{mi}\boldsymbol{\beta}_m$ and $\boldsymbol{\mathcal{V}}\left(\boldsymbol{y}_i\right) = \boldsymbol{\Sigma}_{mi} = \boldsymbol{Z}_i\boldsymbol{\Sigma}_{\boldsymbol{d}}\boldsymbol{Z}_i' + \boldsymbol{\Sigma}_{\boldsymbol{\mathfrak{e}}_{mi}}$. For independent sampling unit i and $\boldsymbol{e}_{mi} = \boldsymbol{Z}_i\boldsymbol{d}_i + \boldsymbol{\mathfrak{e}}_{mi} \sim \mathcal{N}\left(\mathbf{0}, \boldsymbol{\Sigma}_{mi}\right)$, the "population-average" form of a mixed model is

$$\boldsymbol{y}_i = \boldsymbol{X}_{mi}\boldsymbol{\beta}_m + \boldsymbol{e}_{mi} \ . \tag{3}$$

Muller and Stewart (2006), Section 5.2, provided explicit notation to write the model equation for all data considered together as

$$\boldsymbol{y}_m = \boldsymbol{X}_m\boldsymbol{\beta}_m + \boldsymbol{e}_m \ . \tag{4}$$

Here $n = \sum_{i=1}^{N} p_i$, $\boldsymbol{y}_m$ and $\boldsymbol{e}_m$ are $n \times 1$, $\boldsymbol{X}_m$ is $n \times q$, $\boldsymbol{y}_m' = \left[\boldsymbol{y}_1' \ \boldsymbol{y}_2' \ \cdots \ \boldsymbol{y}_N'\right]$, $\boldsymbol{e}_m' = \left[\boldsymbol{e}_{m,1}' \ \boldsymbol{e}_{m,2}' \ \cdots \ \boldsymbol{e}_{m,N}'\right]$ and $\boldsymbol{X}_m' = \left[\boldsymbol{X}_1' \ \boldsymbol{X}_2' \ \cdots \ \boldsymbol{X}_N'\right]$. Also, $\boldsymbol{e}_m \sim \mathcal{N}_n\left(\mathbf{0}_n, \bigoplus_{i=1}^{N} \boldsymbol{\Sigma}_{m,i}\right)$. With no missing data, $p_i \equiv p_{i'}$, $\boldsymbol{\Sigma}_{m,i} \equiv \boldsymbol{\Sigma}_{m,i'}$, and $\bigoplus_{i=1}^{N} \boldsymbol{\Sigma}_{mi} = \boldsymbol{I}_N \otimes \boldsymbol{\Sigma}_{m,i}$.

A general linear mixed model hypothesis about fixed effects can be written in terms of $\boldsymbol{\theta}_m = \boldsymbol{C}_m\boldsymbol{\beta}_m$ as

$$H_{0m} : \boldsymbol{\theta}_m = \boldsymbol{\theta}_{0m} \ . \tag{5}$$

The multivariate general linear model, and its special case the univariate general linear model, is customarily written to describe all observations in the sample:

$$\boldsymbol{Y} = \boldsymbol{X}_M \boldsymbol{B}_M + \boldsymbol{E} \ , \tag{6}$$

with N rows corresponding to independent sampling units, p columns in $\boldsymbol{Y}$, $\boldsymbol{B}$ and $\boldsymbol{E}$ and q columns in $\boldsymbol{X}_M$. Here $\text{row}_i(\boldsymbol{E})' \sim \mathcal{N}_p(\boldsymbol{0}, \boldsymbol{\Sigma}_M)$. Fixed, conforming and known constant contrast matrices $\boldsymbol{C}_M$ and $\boldsymbol{U}$ define the secondary parameter matrix $\boldsymbol{\Theta}_M = \boldsymbol{C}_M \boldsymbol{B}_M \boldsymbol{U}$. For fixed, conforming and known constant matrix $\boldsymbol{\Theta}_{0M}$, the multivariate general linear hypothesis is

$$H_{0M} : \boldsymbol{C}_M \boldsymbol{B}_M \boldsymbol{U} = \boldsymbol{\Theta}_{0M} \ . \tag{7}$$

Assuming $\boldsymbol{X}_M$ is fixed and known without appreciable error and the type I error rate is α, six matrices suffice to fully specify a multivariate power analysis: $\boldsymbol{X}_M$, $\boldsymbol{\Sigma}_M$, $\boldsymbol{C}_M$, $\boldsymbol{B}_M$, $\boldsymbol{U}$ and $\boldsymbol{\Theta}_{0M}$. All $\boldsymbol{X}_M$ are full rank of q, which implies the univariate error degrees of freedom are $\nu_e = N - \text{rank}(\boldsymbol{X}_M) = N - q$.

It is convenient to consider the essence matrix, $\text{Es}(\boldsymbol{X}_M)$, which is created by deleting any duplicate rows in $\boldsymbol{X}_M$. With N_g the replication factor, the number of independent sampling units for each row of $\text{Es}(\boldsymbol{X}_M)$, without loss of generality, for a balanced design $\boldsymbol{X}_M = \text{Es}(\boldsymbol{X}_M) \otimes \boldsymbol{1}_{N_g}$. With G rows in $\text{Es}(\boldsymbol{X}_M)$, a balanced design has a total sample size of $N = G \cdot N_g$. An unbalanced designs has N_g varying across rows of $\text{Es}(\boldsymbol{X}_M)$ and $N = \sum_{g=1}^{G} N_g$ specifies the total sample size (total number of independent sampling units).

1.2 Features Common to All Examples

All examples share the following assumptions. 1) The number of independent sampling units is the same for all levels of any effect that varies between independent sampling units. 2) All clusters are of equal size. 3) There are equal numbers of sub-clusters at each level of nesting. 4) The only predictors are fixed and known indicator variables. 5) Reference cell coding (Muller & Fetterman, 2003) is used. 6) Every independent sampling unit has the same covariance pattern for all observations.

All power values were calculated with the software POWERLIB (Johnson et al., 2009). Code is freely available at *https://www.SampleSizeShop.org*). POWERLIB is open source code that runs in SAS/IML©. Each program defined a "BETASCAL" vector, $\boldsymbol{b} = \begin{bmatrix} 0 & 1 & 2 & \cdots & d \end{bmatrix}' / d$. POWERLIB computes a power value for each choice of $\boldsymbol{B} \cdot b_k$. Choosing $d \approx 100$ provided enough resolution to give smooth plots. In the mathematics in Online Resource 1, δ reflects BETASCAL.

The cluster dimensions are, from innermost to outermost, $\boldsymbol{k}' = \begin{bmatrix} k_1 & k_2 & k_3 \end{bmatrix} = \begin{bmatrix} 6 & 6 & 8 \end{bmatrix}$, corresponding to participants nested in providers, providers nested in clinics, and clinics nested in hospitals. Corresponding intraclass correlation parameters for the variance component model (Longford, 1987) are $\boldsymbol{\rho}' = \begin{bmatrix} \rho_1 & \rho_2 & \rho_3 \end{bmatrix} = \begin{bmatrix} 0.05 & 0.01 & 0.01 \end{bmatrix}$. With $k_* = k_1 \cdot k_2 \cdot k_3$ and $\tau_e = 1 - (\rho_1 + \rho_2 + \rho_3)$, the $k_* \times k_*$ covariance matrix for

all observations from a single hospital is

$$\boldsymbol{\Sigma}_A = \rho_3 \mathbf{1}_{k_*} \mathbf{1}'_{k_*} + \rho_2 \boldsymbol{I}_{k_3} \otimes \mathbf{1}_{k_2 k_1} \mathbf{1}'_{k_2 k_1} + \rho_1 \boldsymbol{I}_{k_3 k_2} \otimes \mathbf{1}_{k_1} \mathbf{1}'_{k_1} + \tau_e \boldsymbol{I}_{k_*} \ . \tag{8}$$

The examples share many, but not all, dimensions. All examples assume 8 outcomes are measured on $k_* = k_1 \cdot k_2 \cdot k_3$ clustered participants for each of 20 hospitals (the independent sampling unit). The subgroup model for question 3 assumes 10 hospitals per subgroup with 5 in each treatment, which gives a total of $N = 10$ independent sampling units. All other models have $N = 20$ independent sampling units. The total number of observations is $n = 8 \cdot k_1 \cdot k_2 \cdot k_3 \cdot N$. Power varies nonlinearly with every dimension, as well as the correlation and variance parameters.

1.3 Reversible Models

With the assumptions, all models for the examples are reversible, as defined by Chi, Glueck, and Muller (2019). A reversible linear model can be stated as either a general linear mixed model or as a general linear multivariate model. The multivariate statement allows using power and sample size methods that give exact results for the examples (Chi et al., 2019; Muller, Lavange, Ramey, & Ramey, 1992) . For the scenarios considered, the Hotelling-Lawley multivariate test statistic is equivalent to the mixed model Wald test statistic (Gurka, Edwards, & Muller, 2011). Chi et al. (2019) provided sufficient conditions to determine whether a mixed model is reversible.

An explicit transformation process allows converting any general linear multivariate model into a general linear mixed model. Equation 12.6 and subsequent expression in Section 12.1 from Muller and Stewart (2006) define the process. Transposing the multivariate model and applying the vec operator gives the data stacked by independent sampling unit:

$$\begin{aligned}
\text{vec}\left(\boldsymbol{Y}'\right) &= \text{vec}\left[(\boldsymbol{X}_M \boldsymbol{B}_M)'\right] + \text{vec}\left(\boldsymbol{E}'\right) \\
\begin{bmatrix} \boldsymbol{Y}'_1 \\ \vdots \\ \boldsymbol{Y}'_N \end{bmatrix} &= \left(\boldsymbol{X}_M \otimes \boldsymbol{I}_p\right) \text{vec}\left(\boldsymbol{B}'_M\right) + \begin{bmatrix} \boldsymbol{E}'_1 \\ \vdots \\ \boldsymbol{E}'_N \end{bmatrix} \\
\begin{bmatrix} \boldsymbol{Y}'_1 \\ \vdots \\ \boldsymbol{Y}'_N \end{bmatrix} &= \begin{bmatrix} \left(\boldsymbol{X}_{M1} \otimes \boldsymbol{I}_p\right) \text{vec}\left(\boldsymbol{B}'_M\right) \\ \vdots \\ \left(\boldsymbol{X}_{MN} \otimes \boldsymbol{I}_p\right) \text{vec}\left(\boldsymbol{B}'_M\right) \end{bmatrix} + \begin{bmatrix} \boldsymbol{E}'_1 \\ \vdots \\ \boldsymbol{E}'_N \end{bmatrix} \\
\boldsymbol{y}_m &= \boldsymbol{X}_m \boldsymbol{\beta}_m + \boldsymbol{e}_m \quad .
\end{aligned} \tag{9}$$

The last form coincides with equation 4, and hence defines a population-average mixed model for all observations. For independent sampling unit i:

$$\begin{aligned}
\boldsymbol{Y}'_i &= \left(\boldsymbol{X}_{Mi} \otimes \boldsymbol{I}_p\right) \text{vec}\left(\boldsymbol{B}'_M\right) + \boldsymbol{E}'_i \\
\boldsymbol{y}_i &= \boldsymbol{X}_{mi} \boldsymbol{\beta}_m + \boldsymbol{e}_{mi} \ .
\end{aligned} \tag{10}$$

Theorem 1.5 in Muller and Stewart (2006) gives

$$\begin{aligned}
\text{vec}(\boldsymbol{\Theta}'_M) &= \text{vec}(\boldsymbol{U}'\boldsymbol{B}'_M\boldsymbol{C}'_M) \\
&= (\boldsymbol{C}_M \otimes \boldsymbol{U}')\text{vec}\left(\boldsymbol{B}'_M\right) \\
\boldsymbol{\theta}_m &= \boldsymbol{C}_m\boldsymbol{\beta}_m \ .
\end{aligned} \tag{11}$$

In summary, a general linear multivariate model and associated multivariate general linear hypothesis may be expressed as a general linear mixed model and associated general linear hypothesis with the following steps. Multivariate model $\boldsymbol{Y} = \boldsymbol{X}_M\boldsymbol{B} + \boldsymbol{E}$ corresponds to the mixed model statement describing all of the data, $\boldsymbol{y}_m = \boldsymbol{X}_m\boldsymbol{\beta}_m + \boldsymbol{e}_m$. Equivalently, $\boldsymbol{y}_{mi} = \boldsymbol{X}_{mi}\boldsymbol{\beta}_m + \boldsymbol{e}_{mi}$ states the mixed model by describing only the data for independent sampling unit i. The multivariate hypothesis $H_{0M} : \boldsymbol{C}_M\boldsymbol{B}_M\boldsymbol{U} = \boldsymbol{\Theta}_{0M}$ corresponds to the general linear hypothesis for the mixed model

$$H_{0m} : \boldsymbol{C}_m\boldsymbol{\beta}_m = \boldsymbol{\theta}_{0m} \ . \tag{12}$$

For all of the data

$$\begin{aligned}
\boldsymbol{y}_m &= \text{vec}\left(\boldsymbol{Y}'\right) \\
\boldsymbol{X}_m &= \boldsymbol{X}_M \otimes \boldsymbol{I}_p \\
\boldsymbol{e}_m &= \text{vec}\left(\boldsymbol{E}'\right) \ .
\end{aligned} \tag{13}$$

For the observations for independent sampling unit i,

$$\begin{aligned}
\boldsymbol{y}_{mi} &= \text{vec}\left(\boldsymbol{Y}'_i\right) \\
&= \left[\text{row}_i\left(\boldsymbol{Y}\right)\right]'
\end{aligned} \tag{14}$$

$$\begin{aligned}
\boldsymbol{X}_{mi} &= \boldsymbol{X}_{Mi} \otimes \boldsymbol{I}_p \\
&= \text{row}_i\left(\boldsymbol{X}_M\right) \otimes \boldsymbol{I}_p
\end{aligned} \tag{15}$$

$$\begin{aligned}
\boldsymbol{e}_{mi} &= \text{vec}\left(\boldsymbol{E}'_i\right) \\
&= \left[\text{row}_i\left(\boldsymbol{E}\right)\right]' \ .
\end{aligned} \tag{16}$$

Here $\boldsymbol{e}_{mi} \sim \mathcal{N}_p\left(\boldsymbol{0}, \boldsymbol{\Sigma}_M\right)$ and $\boldsymbol{e}_m \sim \mathcal{N}_{N\cdot p}\left(\boldsymbol{0}, \boldsymbol{I}_N \otimes \boldsymbol{\Sigma}_M\right)$. For either model statement

$$\begin{aligned}
\boldsymbol{\beta}_m &= \text{vec}\left(\boldsymbol{B}'_M\right) \\
\boldsymbol{C}_m &= \left(\boldsymbol{C}_M \otimes \boldsymbol{U}'\right) \\
\boldsymbol{\theta}_m &= \text{vec}(\boldsymbol{\Theta}'_M) \\
\boldsymbol{\theta}_{0m} &= \text{vec}(\boldsymbol{\Theta}'_{0M}) \ .
\end{aligned} \tag{17}$$

1.4 Question 1 (Figure 2): Does Power Vary with Randomization Level?

Each level of randomization requires a distinct multivariate model. In all models, δ is the intervention effect. For randomization level 1) participant, 2) provider, 3) clinic,

or 4) hospital, the corresponding models are

$$\boldsymbol{Y} = \left(\begin{bmatrix}1\end{bmatrix} \otimes \mathbf{1}_{20}\right)\left(\begin{bmatrix}\delta & \mathbf{0}'_7\end{bmatrix} \otimes \mathbf{1}'_{k_3} \otimes \mathbf{1}'_{k_2} \otimes \begin{bmatrix}\mathbf{0}'_{k_1/2} & \mathbf{1}'_{k_1/2}\end{bmatrix}\right) + \boldsymbol{E} \tag{18}$$

$$\boldsymbol{Y} = \left(\begin{bmatrix}1\end{bmatrix} \otimes \mathbf{1}_{20}\right)\left(\begin{bmatrix}\delta & \mathbf{0}'_7\end{bmatrix} \otimes \mathbf{1}'_{k_3} \otimes \begin{bmatrix}\mathbf{0}'_{k_2/2} & \mathbf{1}'_{k_2/2}\end{bmatrix} \otimes \mathbf{1}'_{k_1}\right) + \boldsymbol{E} \tag{19}$$

$$\boldsymbol{Y} = \left(\begin{bmatrix}1\end{bmatrix} \otimes \mathbf{1}_{20}\right)\left(\begin{bmatrix}\delta & \mathbf{0}'_7\end{bmatrix} \otimes \begin{bmatrix}\mathbf{0}'_{k_3/2} & \mathbf{1}'_{k_3/2}\end{bmatrix} \otimes \mathbf{1}'_{k_2} \otimes \mathbf{1}'_{k_1}\right) + \boldsymbol{E} \tag{20}$$

$$\boldsymbol{Y} = \left(\begin{bmatrix}1 & 0\\ 0 & 1\end{bmatrix} \otimes \mathbf{1}_{10}\right)\left(\begin{bmatrix}0\\ 1\end{bmatrix} \otimes \begin{bmatrix}\delta & \mathbf{0}'_7\end{bmatrix} \otimes \mathbf{1}'_{k_3} \otimes \mathbf{1}'_{k_2} \otimes \mathbf{1}'_{k_1}\right) + \boldsymbol{E}\ . \tag{21}$$

For question 1, levels 1-3 have between contrast matrix $\boldsymbol{C}_{M1,1} = \boldsymbol{C}_{M1,2} = \boldsymbol{C}_{M1,3} = \begin{bmatrix}1\end{bmatrix}$ and level 4 has $\boldsymbol{C}_{M1,4} = \begin{bmatrix}0 & 1\end{bmatrix}$. The exact Hotelling-Lawley test statistic is referenced to an F distribution with degrees of freedom $\{1, 19\}$ for levels 1-3 and $\{1, 18\}$ for level 4. The within-independent sampling unit contrast matrix varies across levels for question 1:

$$\boldsymbol{u}_{1,1} = (\mathbf{1}_8/8) \otimes \mathbf{1}_{k_3} \otimes \mathbf{1}_{k_2} \otimes \begin{bmatrix}-\mathbf{1}_{k_1/2}\\ \mathbf{1}_{k_1/2}\end{bmatrix}(2/k_*) \tag{22}$$

$$\boldsymbol{u}_{1,2} = (\mathbf{1}_8/8) \otimes \mathbf{1}_{k_3} \otimes \begin{bmatrix}-\mathbf{1}_{k_2/2}\\ \mathbf{1}_{k_2/2}\end{bmatrix} \otimes \mathbf{1}_{k_1}\,(2/k_*) \tag{23}$$

$$\boldsymbol{u}_{1,3} = (\mathbf{1}_8/8) \otimes \begin{bmatrix}-\mathbf{1}_{k_3/2}\\ \mathbf{1}_{k_3/2}\end{bmatrix} \otimes \mathbf{1}_{k_2} \otimes \mathbf{1}_{k_1}\,(2/k_*) \tag{24}$$

$$\boldsymbol{u}_{1,4} = (\mathbf{1}_8/8) \otimes \mathbf{1}_{k_3} \otimes \mathbf{1}_{k_2} \otimes \mathbf{1}_{k_1}/k_*\ . \tag{25}$$

In all cases, $\boldsymbol{\Theta}_{M1,L} = \begin{bmatrix}\delta/8\end{bmatrix}$ and $\boldsymbol{\Theta}_{0M1,L} = \begin{bmatrix}0\end{bmatrix}$. The value reflects the assumption of a single composite outcome and the alternative hypothesis that only 1 of 8 of the variables in the composite respond to the intervention.

Since all multivariate models are reversible, equation 9 allows describing corresponding population average mixed models using the following expressions:

$$\begin{aligned}\operatorname{vec}\left(\boldsymbol{Y}'\right) &= \left(\boldsymbol{X}_{M1} \otimes \boldsymbol{I}_p\right)\operatorname{vec}\left(\boldsymbol{B}'_{M1}\right) + \operatorname{vec}\left(\boldsymbol{E}'\right)\\ \boldsymbol{y}_{m1} &= \left(\boldsymbol{X}_{M1} \otimes \boldsymbol{I}_p\right)\operatorname{vec}\left(\boldsymbol{B}'_{M}\right) + \boldsymbol{e}_{m1}\\ \boldsymbol{y}_{m1} &= \boldsymbol{X}_{m1}\boldsymbol{\beta}_{m1} + \boldsymbol{e}_{m1}\ .\end{aligned} \tag{26}$$

For randomization level 1) participant, 2) provider, 3) clinic, or 4) hospital,

$$\boldsymbol{X}_{m1,1}\boldsymbol{\beta}_{m1,1} = \left(\mathbf{1}_{20} \otimes \boldsymbol{I}_{k_1k_2k_3}\right)\left(\begin{bmatrix}\delta\\ \mathbf{0}_7\end{bmatrix} \otimes \begin{bmatrix}\mathbf{0}_{k_3/2}\\ \mathbf{1}_{k_3/2}\end{bmatrix} \otimes \mathbf{1}_{k_2} \otimes \mathbf{1}_{k_1}\right) \tag{27}$$

$$\boldsymbol{X}_{m1,2}\boldsymbol{\beta}_{m1,2} = \left(\mathbf{1}_{20} \otimes \boldsymbol{I}_{k_1k_2k_3}\right)\left(\begin{bmatrix}\delta\\ \mathbf{0}_7\end{bmatrix} \otimes \mathbf{1}_{k_3} \otimes \begin{bmatrix}\mathbf{0}_{k_2/2}\\ \mathbf{1}_{k_2/2}\end{bmatrix} \otimes \mathbf{1}_{k_1}\right) \tag{28}$$

$$\boldsymbol{X}_{m1,3}\boldsymbol{\beta}_{m1,3} = \left(\mathbf{1}_{20} \otimes \boldsymbol{I}_{k_1k_2k_3}\right)\left(\begin{bmatrix}\delta\\ \mathbf{0}_7\end{bmatrix} \otimes \begin{bmatrix}\mathbf{0}_{k_3/2}\\ \mathbf{1}_{k_3/2}\end{bmatrix} \otimes \mathbf{1}_{k_2} \otimes \mathbf{1}_{k_1}\right) \tag{29}$$

$$\boldsymbol{X}_{m1,4}\boldsymbol{\beta}_{m1,4} = \left(\begin{bmatrix}\mathbf{1}_{10} & \mathbf{0}_{10}\\ \mathbf{0}_{10} & \mathbf{1}_{10}\end{bmatrix} \otimes \boldsymbol{I}_{k_1k_2k_3}\right)\left(\begin{bmatrix}0\\ \mathbf{0}_7\\ \delta\\ \mathbf{0}_7\end{bmatrix} \otimes \mathbf{1}_{k_*}\right) . \quad (30)$$

The last equation uses the equivalence

$$\begin{aligned}\left(\begin{bmatrix}0\\1\end{bmatrix} \otimes \begin{bmatrix}\delta & \mathbf{0}_7'\end{bmatrix} \otimes \mathbf{1}_{k_1k_2k_3}\right)' &= \begin{bmatrix}0 & 1\end{bmatrix} \otimes \begin{bmatrix}\delta\\ \mathbf{0}_7\end{bmatrix} \otimes \mathbf{1}_{k_*} \quad (31)\\ &= \begin{bmatrix}0 & \delta\\ \mathbf{0}_7 & \mathbf{0}_7\end{bmatrix} \otimes \mathbf{1}_{k_*}\\ &= \begin{bmatrix}\mathbf{0}_{k_*} & \delta\mathbf{1}_{k_*}\\ \mathbf{0}_{7k_*} & \mathbf{0}_{7k_*}\end{bmatrix} .\end{aligned}$$

The mixed model contrast matrices for randomization at the level of participant, provider, clinic or hospital, are

$$\boldsymbol{C}_{m1,1} = \begin{bmatrix}1\end{bmatrix} \otimes \boldsymbol{u}_{1,1}' \quad (32)$$

$$\boldsymbol{C}_{m1,2} = \begin{bmatrix}1\end{bmatrix} \otimes \boldsymbol{u}_{1,2} \quad (33)$$

$$\boldsymbol{C}_{m1,3} = \begin{bmatrix}1\end{bmatrix} \otimes \boldsymbol{u}_{1,3}' \quad (34)$$

$$\boldsymbol{C}_{m1,4} = \begin{bmatrix}0 & 1\end{bmatrix} \otimes \boldsymbol{u}_{1,4} . \quad (35)$$

In all cases $\boldsymbol{\theta}_{m1,L} = \begin{bmatrix}\delta/8\end{bmatrix}$. All mixed models have

$$\boldsymbol{e}_{mi} \sim \mathcal{N}_{m_*}\left(\mathbf{0}, \boldsymbol{I}_8 \otimes \boldsymbol{\Sigma}_A\right) . \quad (36)$$

1.5 Question 2 (Figure 3): Do Composite and Multivariate Tests Differ in Power?

The example randomizes at the level of the independent sampling unit (Hospital), a group-randomized design, and has no subgroups. Answering the question compares two distinct tests within a single model. Using reference cell coding, the multivariate model is

$$\begin{aligned}\boldsymbol{Y} &= \boldsymbol{X}_M\boldsymbol{B} + \boldsymbol{E} \quad (37)\\ \boldsymbol{Y} &= \left(\begin{bmatrix}1 & 0\\ 1 & 1\end{bmatrix} \otimes \mathbf{1}_{10}\right)\left(\begin{bmatrix}0\\1\end{bmatrix} \otimes \begin{bmatrix}\delta & \mathbf{0}_7'\end{bmatrix} \otimes \mathbf{1}_{k_*}'\right) + \boldsymbol{E}\end{aligned}$$

with $[\text{row}_i(\boldsymbol{Y}_2)]' \sim \mathcal{N}_{8\cdot k_*}(\mathbf{0}, \boldsymbol{I}_8 \otimes \boldsymbol{\Sigma}_A)$. Both tests use between contrast $\boldsymbol{C}_{M2} = \begin{bmatrix}0 & 1\end{bmatrix}$. The within contrasts are for composite

$$\boldsymbol{U}_{2C} = (\mathbf{1}_8/8) \otimes \mathbf{1}_{k_*}/k_* , \quad (38)$$

and for multivariate

$$\boldsymbol{U}_{2M} = \boldsymbol{I}_8 \otimes \mathbf{1}_{k_*}/k_* \ , \tag{39}$$

with degrees of freedom $\{1, 18\}$ for the composite F and $\{1, 11\}$ for the multivariate F. Also, $\boldsymbol{\Theta}_{2C} = \begin{bmatrix}\delta/8\end{bmatrix}$ and $\boldsymbol{\Theta}_{2M} = \begin{bmatrix}\delta \ 0 \ 0 \ 0 \ 0 \ 0 \ 0 \ 0\end{bmatrix}$.

The corresponding mixed model is, with $p = k_* = k_1k_2k_3$,

$$\begin{aligned} \text{vec}\left(\boldsymbol{Y}'\right) &= \left(\boldsymbol{X}_{M2} \otimes \boldsymbol{I}_p\right) \text{vec}\left(\boldsymbol{B}'_{M2}\right) + \text{vec}\left(\boldsymbol{E}'\right) \\ \boldsymbol{y}_{m2} &= \left(\boldsymbol{X}_{M2} \otimes \boldsymbol{I}_p\right) \text{vec}\left(\boldsymbol{B}'_{M2}\right) + \boldsymbol{e}_{m2} \\ \boldsymbol{y}_{m2} &= \boldsymbol{X}_{m2}\boldsymbol{\beta}_{m2} + \boldsymbol{e}_{m2} \ , \end{aligned} \tag{40}$$

for $\boldsymbol{e}_{mi2} \sim \mathcal{N}_{8 \cdot k_*}\left(\mathbf{0}, \boldsymbol{I}_8 \otimes \boldsymbol{\Sigma}_A\right)$. Also

$$\boldsymbol{X}_{m2} = \begin{bmatrix} \mathbf{1}_{10} & \mathbf{0}_{10} \\ \mathbf{1}_{10} & \mathbf{1}_{10} \end{bmatrix} \otimes \boldsymbol{I}_{k_*} \ , \tag{41}$$

$$\begin{aligned} \boldsymbol{\beta}_{m2} &= \text{vec}\left(\boldsymbol{B}'_{M2}\right) \\ &= \text{vec}\left(\begin{bmatrix}0 \ 1\end{bmatrix} \otimes \begin{bmatrix} \delta \\ \mathbf{0}_7 \end{bmatrix} \otimes \mathbf{1}_{k_*}\right) \\ &= \begin{bmatrix} \mathbf{0}_{k_*} \\ \mathbf{0}_{7k_*} \\ \delta\mathbf{1}_{k_*} \\ \mathbf{0}_{7k_*} \end{bmatrix} = \begin{bmatrix} 0 \\ \mathbf{0}_7 \\ \delta \\ \mathbf{0}_7 \end{bmatrix} \otimes \mathbf{1}_{k_*} \ . \end{aligned} \tag{42}$$

The mixed model contrasts are for composite

$$\boldsymbol{C}_{m2C} = \begin{bmatrix}0 \ 1\end{bmatrix} \otimes \left[(\mathbf{1}_8/8) \otimes \mathbf{1}_{k_*}/k_*\right]' \tag{43}$$

and for multivariate

$$\boldsymbol{C}_{m2M} = \begin{bmatrix}0 \ 1\end{bmatrix} \otimes \left(\boldsymbol{I}_8 \otimes \mathbf{1}_{k_*}/k_*\right)'_* \ , \tag{44}$$

which imply $\boldsymbol{\theta}_{m2M} = \begin{bmatrix}\delta/8\end{bmatrix}$ and $\boldsymbol{\theta}_{m2C} = \begin{bmatrix}\delta \ 0 \ 0 \ 0 \ 0 \ 0 \ 0 \ 0\end{bmatrix}'$.

1.6 Question 3 (Figure 4): Do Subgroup and Pooled Analyses Differ in Power?

The multivariate models are for a subgroup

$$\boldsymbol{Y} = \left(\begin{bmatrix} 1 & 0 \\ 1 & 1 \end{bmatrix} \otimes \mathbf{1}_5\right)\left(\begin{bmatrix} 0 \\ 1 \end{bmatrix} \otimes \begin{bmatrix}\delta \ \mathbf{0}'_7\end{bmatrix} \otimes \mathbf{1}'_{k_*}\right) + \boldsymbol{E} \tag{45}$$

and for pooled data

$$\boldsymbol{Y} = \left(\begin{bmatrix} 1 & 0 \\ 1 & 1 \end{bmatrix} \otimes \begin{bmatrix} 1 & 0 \\ 1 & 1 \end{bmatrix} \otimes \mathbf{1}_5\right)\left(\begin{bmatrix} 0 \\ 1 \\ 0 \\ 0 \end{bmatrix} \otimes \begin{bmatrix}\delta \ \mathbf{0}'_7\end{bmatrix} \otimes \mathbf{1}'_{k_*}\right) + \boldsymbol{E} \ , \tag{46}$$

with $[\text{row}_i(\boldsymbol{Y}_2)]' \sim \mathcal{N}_{8\cdot k_*}(\boldsymbol{0}, \boldsymbol{I}_8 \otimes \boldsymbol{\Sigma}_A)$. Between contrasts are

$$\boldsymbol{C}_{M3S} = \begin{bmatrix} 0 & 1 \end{bmatrix} \tag{47}$$

for subgroup and

$$\boldsymbol{C}_{M3P} = \begin{bmatrix} 0 & 1 & 0 & 1/2 \end{bmatrix} \tag{48}$$

for pooled. Both use $\boldsymbol{U}_3 = (\boldsymbol{1}_8/8) \otimes \boldsymbol{1}_{k_*}/k_*$, which implies $\boldsymbol{\Theta}_{M3} = \begin{bmatrix} \delta/8 \end{bmatrix}$. The corresponding F tests have $\{1, 8\}$ and $\{1, 18\}$ degrees of freedom.

The corresponding mixed model design matrices are

$$\boldsymbol{X}_{m3S} = \left(\begin{bmatrix} 1 & 0 \\ 1 & 1 \end{bmatrix} \otimes \boldsymbol{1}_5 \right) \otimes \boldsymbol{1}_{k_*} \tag{49}$$

$$\boldsymbol{X}_{m3P} = \left(\begin{bmatrix} 1 & 0 \\ 1 & 1 \end{bmatrix} \otimes \begin{bmatrix} 1 & 0 \\ 1 & 1 \end{bmatrix} \otimes \boldsymbol{1}_5 \right) \otimes \boldsymbol{1}_{k_*} \ . \tag{50}$$

The parameter matrices are

$$\boldsymbol{\beta}_{m3S} = \begin{bmatrix} 0 \\ 1 \end{bmatrix} \otimes \begin{bmatrix} \delta \\ \boldsymbol{0}_7 \end{bmatrix} \otimes \boldsymbol{1}_{k_*} \ . \tag{51}$$

$$\boldsymbol{\beta}_{m3P} = \begin{bmatrix} 0 \\ 1 \\ 0 \\ 0 \end{bmatrix} \otimes \begin{bmatrix} \delta \\ \boldsymbol{0}_7 \end{bmatrix} \otimes \boldsymbol{1}_{k_*} . \tag{52}$$

The mixed model contrasts are

$$\boldsymbol{C}_{m3S} = \begin{bmatrix} 0 & 1 \end{bmatrix} \otimes (\boldsymbol{1}_8'/8) \otimes \boldsymbol{1}_{k_*}'/k_* \tag{53}$$

$$\boldsymbol{C}_{m3P} = \begin{bmatrix} 0 & 1 & 0 & 1/2 \end{bmatrix} \otimes (\boldsymbol{1}_8'/8) \otimes \boldsymbol{1}_{k_*}'/k_* \ , \tag{54}$$

which both imply $\boldsymbol{\theta}_{m3} = [\delta]$. Both mixed models have $\boldsymbol{e}_{mi3} \sim \mathcal{N}_{8\cdot k_*}(\boldsymbol{0}, \boldsymbol{I}_8 \otimes \boldsymbol{\Sigma}_A)$.

1.7 Power Equivalent Multivariate Models

For computational convenience and ease of understanding, actual calculations were conducted with power equivalent models. Post-multiplying a multivariate model $\boldsymbol{Y} = \boldsymbol{X}_M\boldsymbol{B}_M + \boldsymbol{E}$ by $\boldsymbol{U}$ $(p \times b)$ creates a power equivalent model (see Section 16.4, Muller and Stewart (2006)):

$$\begin{aligned} \boldsymbol{YU} &= \boldsymbol{X}_M\boldsymbol{B}_M\boldsymbol{U} + \boldsymbol{EU} \\ \boldsymbol{Y}_H &= \boldsymbol{X}_M\boldsymbol{B}_H + \boldsymbol{E}_H \ . \end{aligned} \tag{55}$$

The model $\boldsymbol{Y} = \boldsymbol{X}_M\boldsymbol{B}_M + \boldsymbol{E}$ tested the hypothesis $H_0 : \boldsymbol{C}_M\boldsymbol{B}_M\boldsymbol{U} = \boldsymbol{\Theta}_{0M}$. The values of $\boldsymbol{C}_M$ and $\boldsymbol{\Theta}_{0M}$ remain the same in the transformed models. Replacing $\boldsymbol{U}$ with $\boldsymbol{I}_b$ defines the power equivalent hypothesis in the transformed model as $H_0 : \boldsymbol{C}_M\boldsymbol{B}_H = \boldsymbol{\Theta}_{0M}$. With $\boldsymbol{\Sigma}_* = \boldsymbol{U}'\boldsymbol{\Sigma}_M\boldsymbol{U}$,in the power equivalent model, $\text{row}_i(\boldsymbol{E}_H)' \sim \mathcal{N}_p(\boldsymbol{0}, \boldsymbol{\Sigma}_*)$.

For Question 1, we considered randomization levels 1) participant, 2) provider, 3) clinic, and 4) hospital. We created power-equivalent models by applying the following transformations:

$$\boldsymbol{T}_1 = (\mathbf{1}_8/8) \otimes \left(\boldsymbol{I}_2 \otimes \mathbf{1}_{k_3/2}\right) \otimes \mathbf{1}_{k_2} \otimes \mathbf{1}_{k_1}/k_* \tag{56}$$

$$\boldsymbol{T}_2 = (\mathbf{1}_8/8) \otimes \mathbf{1}_{k_3} \otimes \left(\boldsymbol{I}_2 \otimes \mathbf{1}_{k_2/2}\right) \otimes \mathbf{1}_{k_1}/k_* \tag{57}$$

$$\boldsymbol{T}_3 = (\mathbf{1}_8/8) \otimes \mathbf{1}_{k_3} \otimes \mathbf{1}_{k_2} \otimes \left(\boldsymbol{I}_2 \otimes \mathbf{1}_{k_1/2}\right)/k_* \tag{58}$$

$$\boldsymbol{T}_4 = \boldsymbol{u}_{1,4} = (\mathbf{1}_8/8) \otimes \mathbf{1}_{k_3} \otimes \mathbf{1}_{k_2} \otimes \mathbf{1}_{k_1}/k_* \ . \tag{59}$$

For levels 1, 2, and 3, the transformed models use $\boldsymbol{U} = \begin{bmatrix} -1 \\ 1 \end{bmatrix}$. For level 4, $\boldsymbol{U} = \begin{bmatrix} 1 \end{bmatrix}$.

Power analyses for Question 2, comparing composite and multivariate tests, used the transformation $\boldsymbol{T}_4$ in equation 59 for the composite test. The multivariate test used $\boldsymbol{T}_{4M} = \boldsymbol{I}_8 \otimes \mathbf{1}_{k_3} \otimes \mathbf{1}_{k_2} \otimes \mathbf{1}_{k_1}/k_*$. The transformed models used $\boldsymbol{U} = \begin{bmatrix} 1 \end{bmatrix}$ for the composite test and $\boldsymbol{U} = \boldsymbol{I}_8$ for the multivariate test. The values of $\boldsymbol{C}_{M2}$ and $\boldsymbol{\Theta}_{0M,2}$ remain the same in the transformed models.

Power analyses for Question 3, subgroup and pooled analysis, used the transformation $\boldsymbol{T}_4$ in equation 59 for both the subgroup and pooled models. The transformed models used

$$\boldsymbol{U} = \begin{bmatrix} 1 \end{bmatrix} \ . \tag{60}$$

The values of $\boldsymbol{C}_{M3}$ and $\boldsymbol{\Theta}_{0M,3}$ remain the same in the transformed models.

References

Chi, Y.-Y., Glueck, D.H., Muller, K.E. (2019). Power and sample size for fixed-effects inference in reversible linear mixed models. *The American Statistician*, *73*(4), 350–359, https://doi.org/10.1080/00031305.2017.1415972

Gurka, M.J., Edwards, L.J., Muller, K.E. (2011). Avoiding bias in mixed model inference for fixed effects. *Statistics in Medicine*, *30*(22), 2696–2707, https://doi.org/10.1002/sim.4293

Johnson, J.L., Muller, K.E., Slaughter, J.C., Gurka, M.J., Gribbin, M.J., Simpson, S.L. (2009). POWERLIB: SAS/IML software for computing power in multivariate linear models. *Journal of Statistical Software*, *30*(5), http://www.jstatsoft.org/v30/i05, https://doi.org/10.18637/jss.v030.i05

Longford, N.T. (1987). A fast scoring algorithm for maximum likelihood estimation in unbalanced mixed models with nested random effects. *Biometrika*, *74*(4), 817–827, https://doi.org/10.2307/2336476

Muller, K.E., & Fetterman, B.A. (2003). *Regression and ANOVA: an integrated approach using SAS software*. John Wiley & Sons, Inc.

Muller, K.E., Lavange, L.M., Ramey, S.L., Ramey, C.T. (1992). Power calculations for general linear multivariate models including repeated measures applications. *Journal of the American Statistical Association*, *87*(420), 1209–1226, https://doi.org/10.1080/01621459.1992.10476281

Muller, K.E., & Stewart, P.W. (2006). *Linear model theory: Univariate, multivariate, and mixed models*. New York, New York: John Wiley & Sons. (Google-Books-ID: bLwRX4S676QC)
