# Supplementary figures and images for: Using Power Analysis to Choose the Unit of Randomization, Outcome, and Approach for Subgroup Analysis for a Multilevel Randomized Controlled Clinical Trial to Reduce Disparities in Cardiovascular Health
Source: Prev Sci. 2024 May 20;25(Suppl 3):433–45. doi: 10.1007/s11121-024-01673-y (PMC11239604; doi:10.1007/s11121-024-01673-y)

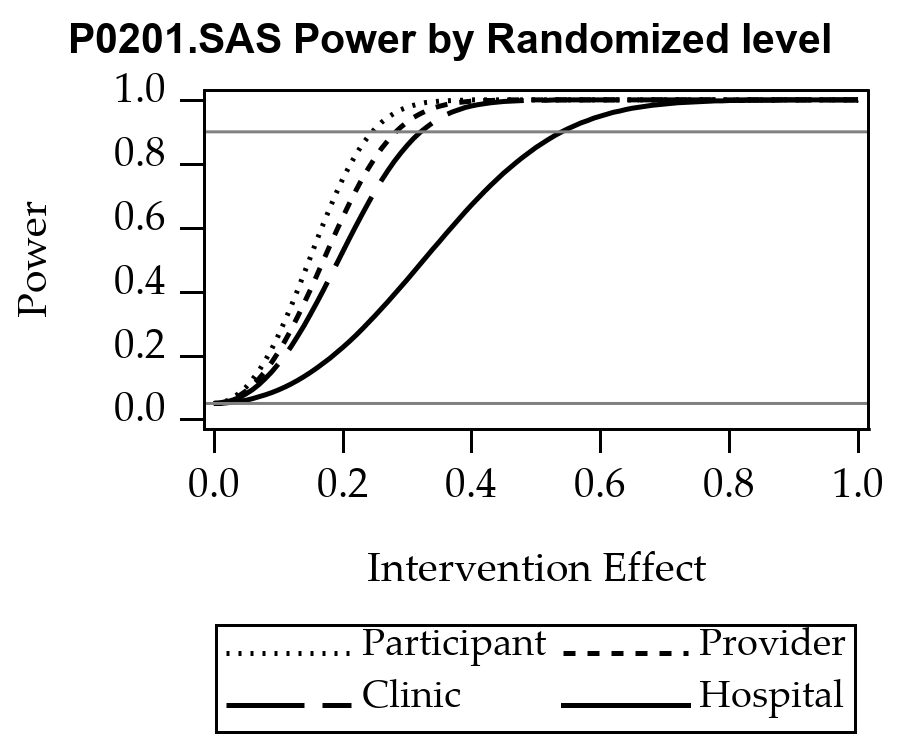

Supplement: Supplementary file 2 — Supplementary file2 (ZIP 303 KB) [file 11121_2024_1673_MOESM2_ESM.zip › P0201.png]

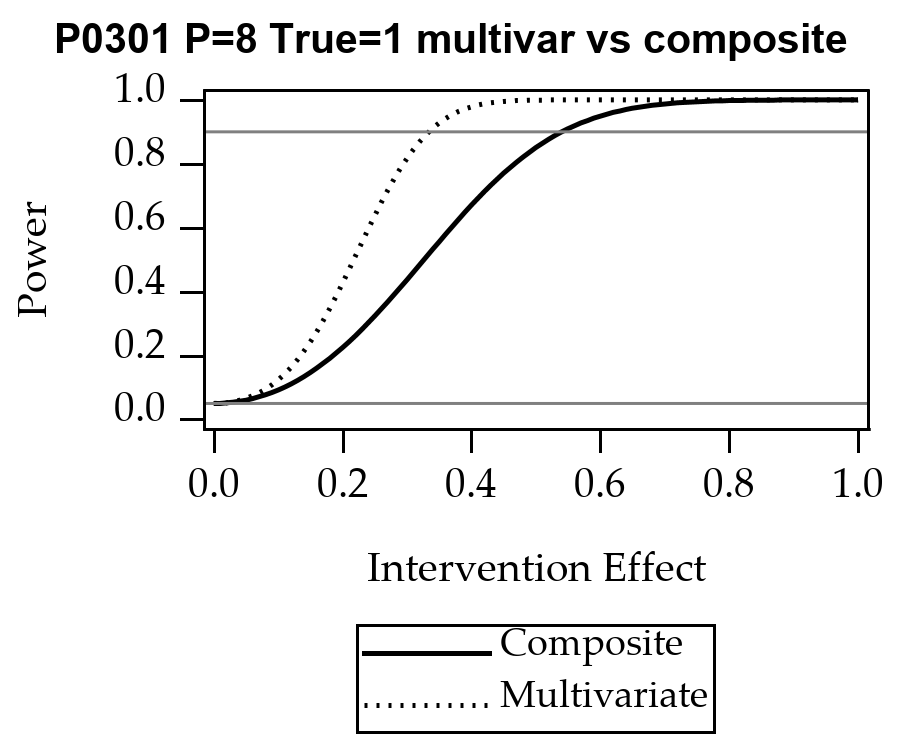

Supplement: Supplementary file 2 — Supplementary file2 (ZIP 303 KB) [file 11121_2024_1673_MOESM2_ESM.zip › P0301.png]

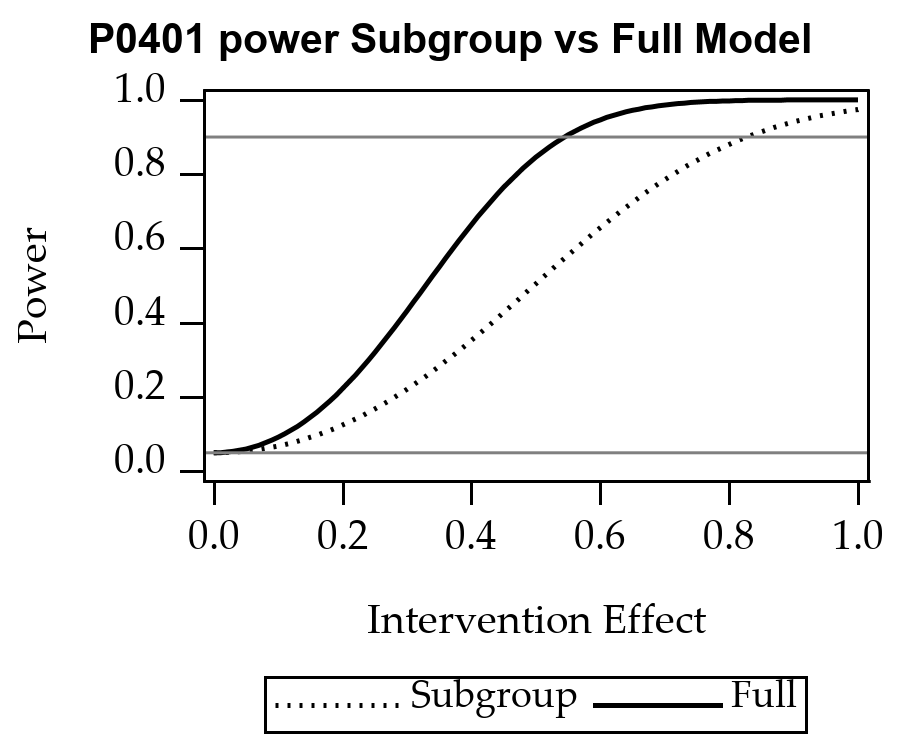

Supplement: Supplementary file 2 — Supplementary file2 (ZIP 303 KB) [file 11121_2024_1673_MOESM2_ESM.zip › P0401.png]
